# Effect of Low Light on Photosynthetic Performance of Tomato Plants—Ailsa Craig and Carotenoid Mutant *Tangerine*

**DOI:** 10.3390/plants12163000

**Published:** 2023-08-20

**Authors:** Maya Velitchkova, Martin Stefanov, Antoaneta V. Popova

**Affiliations:** Institute of Biophysics and Biomedical Engineering, Bulgarian Academy of Sciences, Acad, G. Bonchev Str. Bl. 21, 1113 Sofia, Bulgaria; martin@bio21.bas.bg (M.S.);

**Keywords:** 77K fluorescence, electron transport rate, fluidity, low light intensity, low temperature, photosystem I, photosystem II, PSII antenna proteins, *tangerine* mutant, tomato

## Abstract

The effects of a five-day treatment with low light intensity on tomato plants—Ailsa Craig and *tangerine* mutant—at normal and low temperatures and after recovery for three days under control conditions were investigated. The *tangerine* tomato, which has orange fruits, yellowish young leaves, and pale blossoms, accumulates prolycopene rather than all-*trans* lycopene. We investigated the impact of low light at normal and low temperatures on the functioning and effectiveness of photosynthetic apparatuses of both plants. The photochemical activities of Photosystem I (PSI) and Photosystem II (PSII) were assessed, and the alterations in PSII antenna size were characterized by evaluating the abundance of PSII-associated proteins Lhcb1, Lhcb2, CP43, and CP47. Alterations in energy distribution and interaction of both photosystems were analyzed using 77K fluorescence. In Aisla Craig plants, an increase in thylakoid membrane fluidity was detected during treatment with low light at a low temperature, while for the *tangerine* mutant, no significant change was observed. The PSII activity of thylakoids from mutant *tangerine* was more strongly inhibited by treatment with low light at a low temperature while low light barely affected PSII in Aisla Craig. The obtained data indicated that the observed differences in the responses of photosynthetic apparatuses of Ailsa Craig and *tangerine* when exposed to low light intensity and suboptimal temperature were mainly related to the differences in sensitivity and antenna complexes of PSII.

## 1. Introduction

The development and productivity of higher plants are dependent on environmental conditions such as light intensity and temperature [1]. Numerous important crops, such as cotton, tomato, maize, rice, and tobacco, are native to tropical regions and require high temperatures and intense sunshine to grow [2]. Development at suboptimal light and temperature conditions seriously reduces biomass production, photosynthetic performance, and yield, as was observed for tomato [3,4], sweet pepper [5], rice [6], and maize [7].

While the effect of high light intensity on photosynthesis has been widely studied, such studies have been lacking in examining the role of low light intensity. The development of plants at lower-than-optimal light intensities suppress not only plant growth but additionally cause significant alterations in plant morphology, such as plant height and specific leaf area, thus leading to a reduction in net photosynthetic rate and respiration [6,8,9]. Low light-tolerant plants respond to low-light treatment with an increase in their chlorophyll b (Chl b) content and a decrease in their Chl a/b ratio [6]. Plants acclimated to growth at low light intensities demonstrate less biomass and increased membrane lipid peroxidation [10]. The low temperature is another key environmental stressor that has a considerable impact on ecosystem abundance and productivity [1,11]. Some tropical species are able to acclimate to suboptimal growth temperatures, but the physiological and molecular mechanisms of cold acclimation are still not completely understood [12]. Recently, it has been shown that different combinations of temperature and light intensity can lead to the mitigation of the negative effect of one or more factors [13]. Low temperature has a more pronounced negative effect on photosynthetic capacity, but the yields can be improved by the combination of low temperature with appropriate light intensity [13].

Carotenoids represent a vast and diverse group of natural pigments. They are substantial components of the photosynthetic apparatus mainly involved in light harvesting, antioxidant, photoprotective, and structural processes [14]. The influence of carotenoid concentration on crop fruit formation has been extensively discussed [15,16,17], but there is little information on how carotenoid content affects plant responses to low light and low temperature.

Tomato (*Solanum lycopersicum* L.) is a photophilous vegetable crop that is frequently cultivated under controlled conditions during winter and spring to meet the increasing demands of humans’ healthy diet. Given that tomato plants are native to tropical locations, their growth and productivity can be significantly reduced by low light and unfavorable temperature conditions [5,18]. However, there are reports that tomato plants can acclimate to cold temperatures at both transcriptomic and metabolic levels [19,20]. The development of tomato plants in weak light and at suboptimal temperature leads to lower activity of PSII and energy dissipation, lower electron transport (ETR) and CO_2_ consumption [3,4,21,22], and disordering of assimilation metabolisms [23]. In addition, a significant reduction in expression of gene-encoding proteins of the PSII reaction center (RC) (psbA, psaB, psbD, psbP, and cab) was detected after 7 days of treatment of tomato plants with low light [3].

Creating different mutants with altered carotenoid content presented a new perspective for unravelling the impact of various carotenoid species on the response of higher plants to extreme environmental conditions. The carotenoid tomato mutant *tangerine* is characterized by defective prolycopene isomerase (CRTISO) that is responsible for the isomerization of tetra-cis-lycopene to all-*trans*-lycopene [24,25], and due to which, tomato accumulates prolycopene instead of all-*trans*-lycopene. The increased study interest in *tangerine* mutants is due to the fact that tetra-*cis* lycopene has roughly 8.5 times higher bioavailability in humans than all-*trans* lycopene [26].

Tomato, being a tropical region plant, is relatively sensitive to suboptimal temperature, and depending on the duration of exposure to low temperatures, some physiological functions such as photosynthesis, respiration, and membrane integrity may be negatively affected [18]. Recently, we reported that the exposure of tomato plants for several days to low day/night temperature and optimal light intensity resulted in changes in pigment content and oxygen evolution from detached leaves [27].

The aim of the present study was to investigate whether and to what extent tomato plants’ responses to low-light and low-temperature treatments were changed by the CRTISO mutation. In order to achieve this, young plants (*Solanum lycopersicum*), wild type Ailsa Craig, and the *tangerine* mutant were all treated for 5 days with a combination of low light (125 mol photons m^−2^ s^−1^) and suboptimal temperature (15/10 °C day/night). The ability of plants to recover after further development for 3 days at normal conditions was also monitored. Low light illumination was chosen based on it being under the optimal light intensity, and in the presence of chlorophyll, it could enable *cis*-*trans* isomerization [24,25]. An investigation was performed on thylakoid membranes, isolated from control, treated, and recovered plants. The effect of exposure to low light at a suboptimal temperature on the photochemical activities and energy distribution and interaction of both photosystems were analyzed in isolated thylakoid membranes from Ailsa Craig and *tangerine*. The changes in physicochemical properties of thylakoid membranes were characterized by the evaluation of membrane fluidity. The alterations of antenna complexes of PSII were estimated by treatment-induced changes in the abundance of Lhcb1 and Lhcb2, and CP43 and CP47.

## 2. Results

Our preliminary experiments with tomato plants showed that the development for 5 days under controlled light intensity and temperature conditions (here and hereafter we will refer to the conditions under which the plants were grown before the treatment as control or normal) did not affect the investigated parameters for the indicated period of time. Our previous investigation [27] has shown that the pigment content and the ratio Fv/Fm for tomato plants (M82) remained unchanged for six days under control conditions. Therefore, and in order not to complicate the presentation, data for plants that were grown under normal conditions for the entire experimental set-up are not included.

### 2.1. Alterations of Pigment Content during Treatment

Data about the content of chlorophyll a (Chl a) and chlorophyll b (Chl b) and total carotenoids (Car) in leaves from control, treated and recovered plants are presented in Table 1. The content of Chl (a+b) and Car seems to be slightly lower in the *tangerine* mutant and ratio Chl a/b was higher, indicating a lower content of Chl b in comparison to Chl a. Exposure of plants for 5 days to low light (LL) at normal temperature (NT) led to a decrease of Chl (a+b) content by 10% for Ailsa Craig and 14% for *tangerine*. After exposure to low-light (LL) and low-temperature (LT) treatment, the decrease was more pronounced—by about 20% for Aisla Craig and *tangerine*. During recovery for 3 days, the Chl (a+b) in plants exposed to LL-NT increased up to 95% and 89% of the controls (0 days) for Ailsa Craig and *tangerine*, respectively. Plants treated with low light and low temperature (LL-LT) did not restore their chlorophyll content to the same extent—Ailsa Craig remained at 79% and mutant at 85% of the control. Car content decreased in *tangerine* and Aisla Craig plants at both temperatures, but the *tangerine*’s decline was more pronounced. During the recovery phase, the Car content in the mutant recovered more effectively than in Ailsa Craig.

### 2.2. Changes of Photosynthetic Performance of Tomato Plants

**Figure 1:**
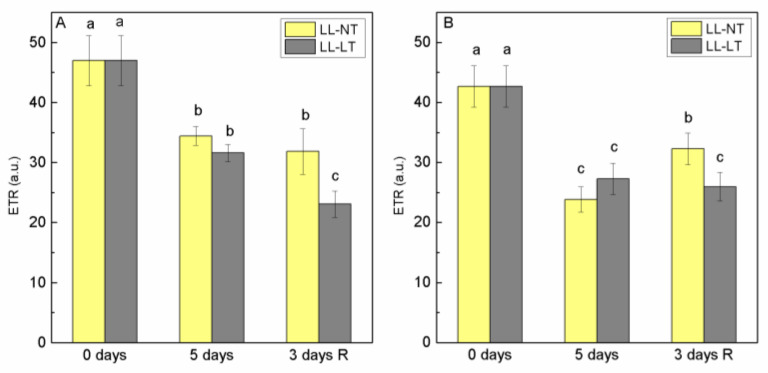
Alterations of values for ETR in thylakoid membranes from Ailsa Craig (**A**) and *tangerine* (**B**) plants, treated with low light (LL) and control (NT) or low (LT) temperature for 5 days and after recovery at control conditions for 3 days. Mean values ± SE were calculated from two independent experiments with 3 parallel samples at every time point (*n* = 6). Significant differences between values at *p* < 0.05, as estimated by Fisher’s LSD test of multifactor ANOVA analysis, were indicated with different letters.

The photosynthetic efficiency of plants exposed to LL at NT or LT and after recovery was evaluated by measuring the maximal PSII quantum efficiency (Fv/Fm) of detached leaves. No significant changes were observed after exposure for 5 days to LT and LL—the ratio was 0.78 ± 0.04 and was close in both Ailsa Craig and *tangerine* plants. During the 3-day recovery period under control conditions, these values were retained (data not shown). To assess the photosynthetic competence of PSII, we evaluated the changes of the relative electron transport rate (ETR). After five days at low light, ETR decreased in Ailsa Craig at both temperatures, and was better expressed at a low temperature (Figure 1A). The ETR for *tangerine* also decreased during exposure to LL, but after the returning of plants to control conditions, a restoration was detected for plants exposed to LL-NT (Figure 1B).

### 2.3. Photochemical Activity of PSI and PSII of Thylakoid Membranes Isolated from Control and Treated Plants

**Figure 2:**
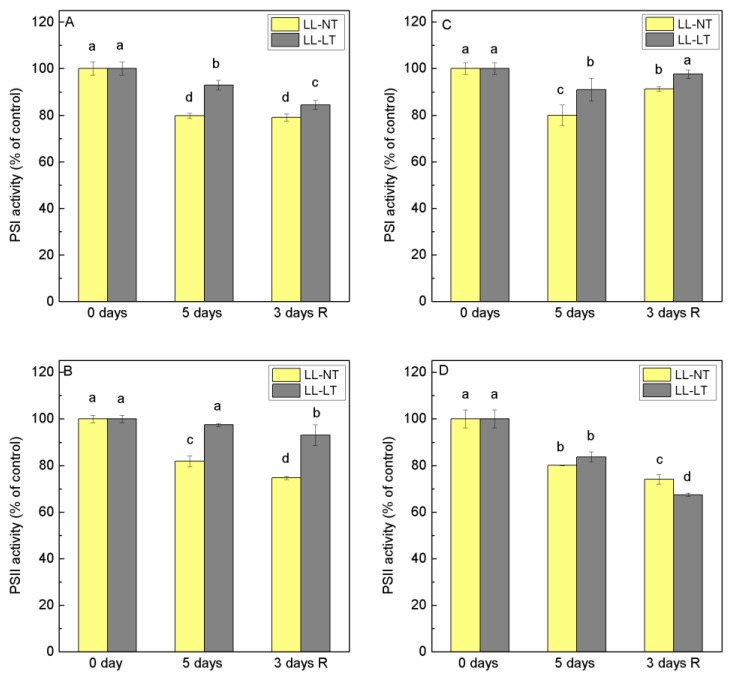
Alterations in photochemical activity of PSI (**A**,**C**) and PSII (**B**,**D**) in thylakoid membranes from Ailsa Craig (**A**,**B**) and *tangerine* (**C**,**D**) plants, treated with low light (LL) and control (NT) or low (LT) temperature for 5 days, and after recovery at control conditions for 3 days. Results were presented as % from the respective, non-treated control plants (0 days). For PSI, 100% corresponded to 91.5 ± 7.2 and 128.3 ± 3.1 µmol O_2_ mg Chl^−1^ h^−1^ for Ailsa Craig and *tangerine*, respectively. For PSII, 100% corresponded to 30.4 ± 1.7 for Ailsa Craig and 48.3 ± 1.8 1 µmol O_2_ mg Chl^−1^ h^−1^ for *tangerine*. Mean values ± SE were calculated from two independent experiments with 3 parallel samples at every time point (*n* = 6). Significant differences between values at *p* < 0.05 as estimated by Fisher’s LSD test of multifactor ANOVA analysis were indicated with different letters.

Chloroplasts are very sensitive organelles to changes in ambient light and temperature, and processes in the thylakoid membranes can be seriously disturbed under unfavorable conditions. In order to evaluate the photochemical activity of both photosystems in thylakoids, the rates of electron transport through PSI and PSII in the presence of exogenous electron acceptors and donors were evaluated. For measurements of PSII-mediated electron transport, we used 1,4 benzoquinone (BQ). For the determination of PSI-mediated electron transport, we applied reduced DCPIP as an electron donor and methyl viologen as an electron acceptor from PSI. DCMU was added to stop electron transfer from PSII. Data of thylakoid membranes isolated from Ailsa Craig and *tangerine* mutant plants at the begging of experiments (0 days) and after 5 days of treatment and after 3 days of recovery are presented in Figure 2. In Ailsa Craig thylakoids, the activity of PSI decreased after 5 days at LL-NT by 20%, while in thylakoids from plants treated with LL-LT, the decline was less expressed—by only 7% (Figure 2A). No recovery was detected after 3 days at control conditions and the activity in LL-LT-treated plants continued to decrease. Although a similar extent of inhibition of PSI was registered for thylakoid membranes of the *tangerine* mutant, a difference was observed during the recovery period—the PSI photochemical activity of LL-NT- as well as of LL-LT-treated plants was restored (Figure 2C).

PSII activity in thylakoids from Ailsa Craig plants treated with LL-NT was inhibited by about 20%, while for those treated with LL-LT, the inhibition was less expressed (Figure 2B). However, for *tangerine* plants, the treatment with LL at NT or LT expressed a significant reduction of PSII photochemistry (by 20 and 18%, respectively) and no recovery occurred after 3 days at control conditions, and an even larger reduction was detected (inhibition by 24 and 30%, for LL-NT and LL-LT, respectively) (Figure 2D).

### 2.4. Effect of Treatment on Parameters of 77K Fluorescence

**Figure 3:**
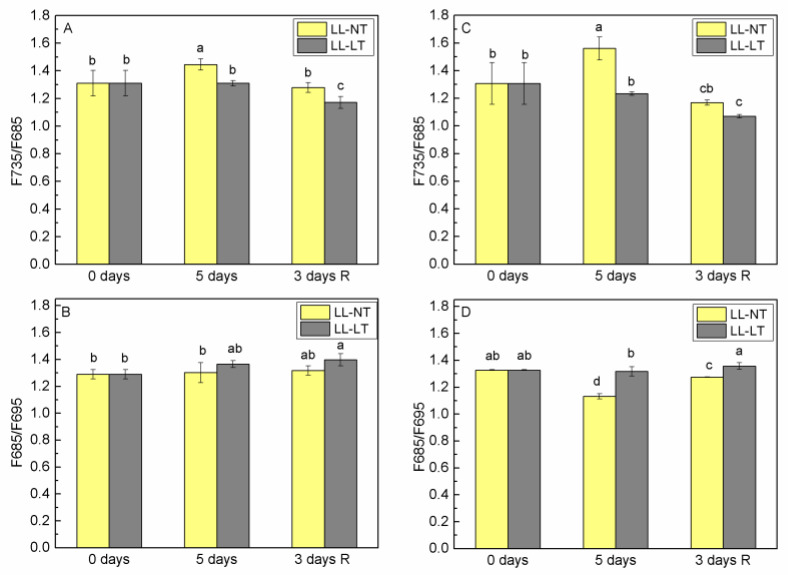
Effect of treatment with low light (LL) at control (NT) or low (LT) temperature and after recovery at control conditions on fluorescence ratio F735/F685 (**A**,**C**) and F685/F695 (**B**,**D**) of Ailsa Craig (**A**,**B**) and *tangerine* (**B**,**D**) thylakoid membranes. Mean values ± SE were calculated from two independent experiments with two parallel samples at every time point (*n* = 4). Significant differences between values at *p* < 0.05, as estimated by Fisher’s LSD test of multifactor ANOVA analysis, were indicated with different letters.

Analysis of low-temperature (77K) fluorescence emission and excitation spectra (Figure S1) provided very useful information about the distribution of excitation energy between photosystems, energy interaction between them and the involvement of Chl a and Chl b molecules in the energy supply of PSI and PSII. Alterations of the ratio F735/F685 as a result of treatment for 5 days with LL-NT and LL-LT and after a 3-day recovery for Ailsa Craig and *tangerine* are presented in Figure 3A,C, respectively. Five-day treatment with LL-NT resulted in an increase of F735/F685 for both plants that was more pronounced for the *tangerine* mutant. After recovery for 3 days at control conditions (NL-NT), the values for Ailsa Craig restored to those for 0 days but decreased in the thylakoids of *tangerine* plants. The ratio F735/F685 did not change significantly after 5 days at LL-LT for Ailsa Craig and *tangerine*, However, after a 3-day recovery period the values were lower for both plants. Stronger expressed difference between Ailsa Craig and *tangerine* was observed for fluorescence emission in the region 685–695 nm (Figure 3B,D). While 5-day treatment with LL-NT did not affect the ratio F685/F695 for Ailsa Craig, for *tangerine*, a well-pronounced decrease was registered, that restored to initial values after a 3-day recovery period. At LL-LT conditions, no significant alterations were detected but slight, not statistically significant increases were observed after recovery for the Ailsa Craig and *tangerine* mutant.

**Figure 4:**
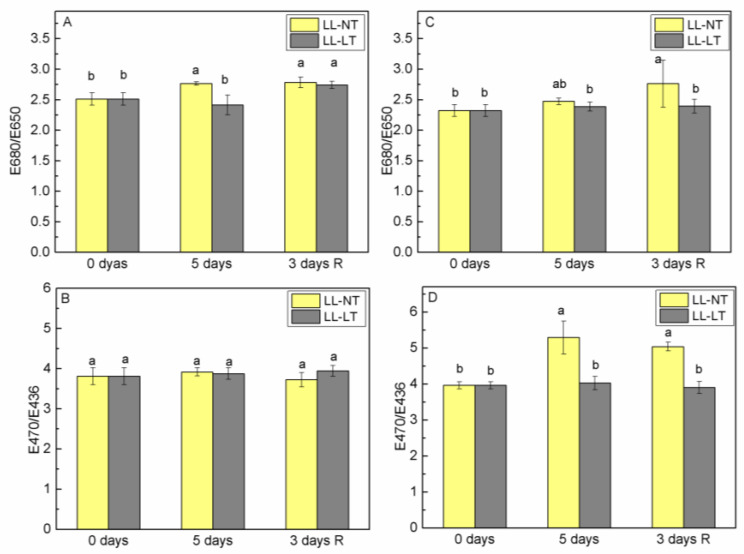
Fluorescence excitation ratios of emission at 735 nm (E680/E650) (**A**,**C**) and at 685 nm (E470/E436) (**B**,**D**) in thylakoid membranes from Ailsa Craig (**A**,**B**) and *tangerine* (**B**,**D**) plants, treated with low light (LL) at control (NT) or low (LT) temperature and after recovery at control conditions. Mean values ± SE were calculated from two independent experiments with two parallel samples at every time point (*n* = 4). Significant differences between values at *p* < 0.05, as estimated by Fisher’s LSD test of multifactor ANOVA analysis, were indicated with different letters.

Analysis of the alterations of the ratio E680/E650 for emission at 735 nm (PSI) and E470/E436 for emission at 685 nm (PSII) allowed for the evaluation of the relative involvement of Chl a and Chl b molecules in the energy supply of PSI and PSII, respectively (Figure 4). Treatment with LL at NT resulted in a well-expressed elevation of the ratio E680/E650 for Ailsa Craig thylakoids (from 2.51 before stress treatment to 2.76), which did not restore after the recovery period (Figure 4A). In Ailsa Craig thylakoids, no statistically significant alterations in the ratio E470/E436 were detected (Figure 4B). Analysis of excitation spectra of thylakoid membranes isolated from treated *tangerine* plants showed that treatment with LL significantly altered the antenna complex of PSII at NT in terms of an increased involvement of Chl b molecules in the energy supply of PSII in comparison with Chl a (Figure 4D). However, when LL was accompanied with LT, no such changes were observed. As for PSI, no statistically significant changes in the E680/E650 ratio were detected in *tangerine* plants after recovery from LL-LT treatment (Figure 4C).

### 2.5. Lipid Order Alterations during Treatment and after Recovery

**Figure 5:**
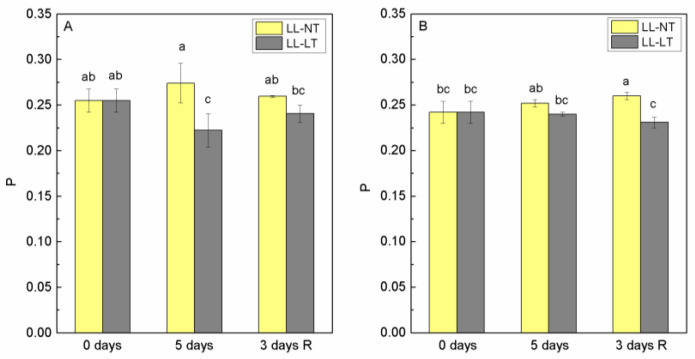
Degree of fluorescence polarization (P) of the fluorescent probe DPH in thylakoid membranes from Ailsa Craig (**A**) and *tangerine* (**B**) plants, treated with low light (LL) at control (NT) or low (LT) temperature and after recovery at optimal conditions. Mean values ± SE were calculated from two independent experiments with at least 3 parallel samples at every time point (*n* = 6). Significant differences between values at *p* < 0.05 were estimated by Fisher’s LSD test of multifactor ANOVA analysis and indicated with different letters.

In order to check if and how a 5-day treatment with low light intensity at normal and low temperature affected the properties of the lipid phase of the thylakoid membrane, we determined the extent of polarization of fluorescence (P) emitted by probe DPH in thylakoid membranes from leaves of tomato plants Ailsa Craig and *tangerine* at the beginning of the experiment—0 days and after 5 days at LL-NT and LL-LT and after 3 days of recovery at control conditions. The values for P are presented in Figure 5. The values for P before treatment were very close and corresponded to the value for plant thylakoid membranes [28]; for Ailsa Craig, this was 0.255 ± 0.013 and 0.242 ± 0.012 for the *tangerine* mutant. After five days of LL-NT treatment, an increase of P was observed in Ailsa Craig, which was within the error limits but after treatment with LL-LT, a decrease in P was found, which recovered after the return of plants to control conditions (Figure 5A). After 5 days of treatment, almost no change in P was detected for thylakoid membranes from the *tangerine* mutant, but a decrease was registered after 3 days recovery (P was 0.231 ± 0.006) for plants treated for 5 days with LL-LT (Figure 5B).

### 2.6. Changes of PSII-LHCII—Associated Polypeptides

**Figure 6:**
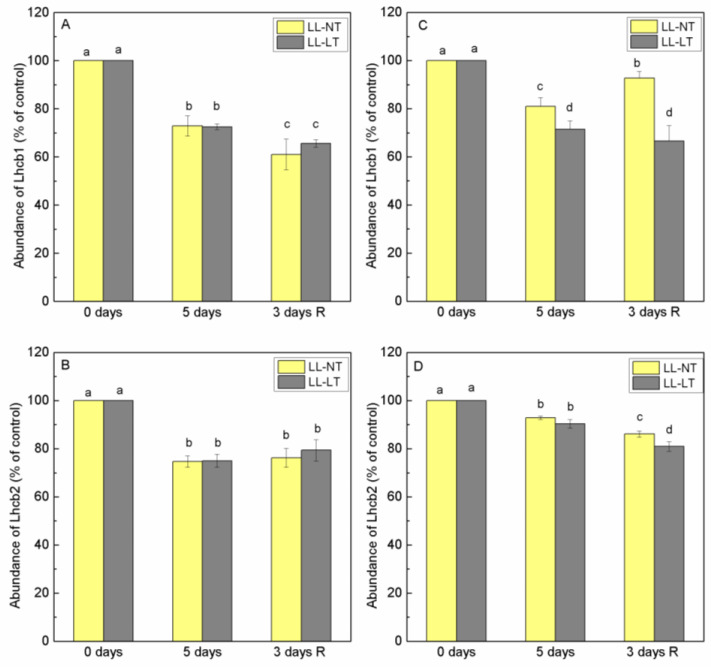
Abundance of Lhcb1 (**A**,**C**) and Lhcb2 (**B**,**D**) in thylakoid membranes from Ailsa Craig (**A**,**B**) and *tangerine* (**C**,**D**) plants, treated with low light (LL) at control (NT) or low (LT) temperature and after recovery at optimal conditions. Results were presented as % from the respective, non-treated control plants (0 days). Mean values ± SE were calculated from two independent experiments with at least 3 parallel samples at every time point (*n* = 6). Significant differences between values at *p* < 0.05 were estimated by Fisher’s LSD test of multifactor ANOVA analysis and indicated with different letters.

**Figure 7:**
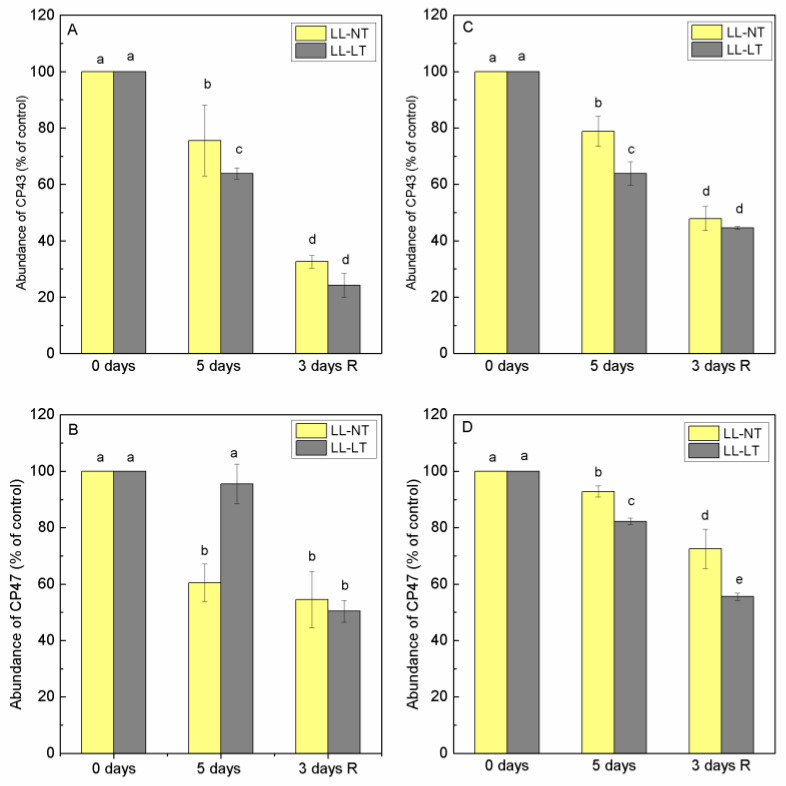
Abundance of CP43 (**A**,**C**) and CP47 (**B**,**D**) in thylakoid membranes from Ailsa Craig (**A**,**B**) and *tangerine* (**C**,**D**) plants, treated with low light (LL) at control (NT) or low (LT) temperature and after recovery at optimal conditions. Results were presented as % from the respective, non-treated control plants (0 days). Mean values ± SE were calculated from two independent experiments with at least 3 parallel samples at every time point (*n* = 6). Significant differences between values at *p* < 0.05 were estimated by Fisher’s LSD test of multifactor ANOVA analysis and indicated with different letters.

The results of fluorescence excitation spectra at 77K showed that there were changes in the ratio E470/E435 ratio of the emission at 685 nm, reflecting the involvement of Chl a and Chl b in the energy supply of PSII. After low-light treatment at normal temperature, an increase in this ratio was recorded and was maintained during the recovery period. To test to what extent this was due to changes in proteins of the nearby antenna (CP43 and CP47) or proteins of LHCII, Lhcb1 and Lhcb2, we analyzed the abundance of these proteins during treatment (Figure S2). Thylakoid membranes from leaves of Ailsa Craig and *tangerine* plants—control (0 days) and treated for 5 days with LL-NT and LL-LT—were examined using SDS-PAGE followed by a Western blot with the respective antibodies. In Ailsa Craig, Lhcb1 content decreased to the same extent at both temperatures and the content of Lhcb2 followed a similar tendency and extent as Lhcb1 (Figure 6A,B). Data showed that in *tangerine*, the amount of Lhcb1 decreased under LL treatment, being stronger expressed at LT and did not recover during the 3-day period under control conditions (Figure 6C). At both temperatures, Lhcb2 in *tangerine* was less impacted than Lhcb1 and the modifications were more subtle. Proximal antenna protein CP43 showed a higher sensitivity in thylakoids from Ailsa Craig, the reduction of content was more pronounced at LT, while for the *tangerine* mutant, the decrease of CP43 was less expressed (Figure 7A,C). No recovery was detected for both plants. After 5 days of treatment, there was a detectable drop in the abundance of CP47 in Ailsa Craig by approximately 40% at LL-NT, but nearly no change in LL-LT-treated plants. For *tangerine*, a gradual decrease in CP47 was detected, which was more evident at low temperatures. During the three-day recovery period, no restoration took place (Figure 7B,D).

## 3. Discussion

Carotenoids are not only a vital part of plants, but also extremely important for human nutrition. Many studies on carotenoid biosynthesis mutations have shown that the content of beneficial carotenoids serving as antioxidants can be adjusted and manipulated. From this point of view, the study of *tangerine* mutants is of interest for both nutritionists and plant physiologists. In addition to having tetra-*cis* lycopene, *tangerine* tomatoes also have comparatively high concentrations of phytoene, phytofluene, α-carotene, and neurosporene in comparison to other tomato cultivars [29]. *Tangerine* mutants have been well described in terms of growth parameters, morphology and carotenoid composition in fruits [24]. The effect of genetic perturbations that impair the function of the CAROTENOID ISOMERASE (CRTISO) on the roots and response to drought in MicroTom *tangerine* (*tangmic*) has been investigated. [30]. However, little is known and data are scarce concerning how and to what extent this mutation influences the photosynthetic apparatus of plants as well as the effectiveness and photochemistry of photosystems and primary photosynthetic reactions related to light perception and transduction.

### 3.1. Pigment Content and Photosynthetic Electron Transport Rate in Leaves of Tomato Plants

In the present study, we compared the response of Ailsa Craig (wild type) and *tangerine* mutant (LA3183) in terms of photochemistry and energy interaction between the two photosystems to low-light-intensity treatment and how this response depended on temperature—normal and suboptimal.

Pigment contents of leaves of control Ailsa Craig and *tangerine* plants differed slightly (Table 1), which is in line with previously released details on this mutant [17]. The *tangerine*’s Chl (a+b) content was found to be lower than that of the wild type, while the mutant’s Chl a/b ratio was higher. It should be mentioned that our assays were performed on 3–3.5-week-old plants, and the leaves used were relatively young. The Chl a/b ratio was barely affected by LL-NT treatment in the wild type, but a drop was detected in the mutant, more noticeable at low temperatures. A relative decrease of Chl a and an increase in Chl b was reported recently for tomato plants treated with different combinations of light intensity and low temperatures [31]. Low-temperature treatment for six days in tomato plants has been shown to result in a decrease in Chla/b ratio [27]. Pigment reduction as a result of exposure to low temperature has been observed in both chilling-resistant and chilling-sensitive plants such as spinach [32], pepper varieties [33], etc. The content of Car was less altered with both treatments in *tangerine* in comparison with Ailsa Craig plants. Low light intensity had little impact on pigments’ concentration at optimal temperature; hence, the effect of LLT-LT treatment was most likely due to the low temperature. The decrease of chlorophyll content during exposure to low and cold temperature were related to a temperature-induced decrease in pigment biosynthesis [34] and/or accelerated pigment degradation as a result of inhibited activities of oxygen radical scavenging enzymes [35].

Our findings for Ailsa Craig and *tangerine* are consistent with newly released information regarding the similar Fv/Fm values in wild type and CRTISO mutant ccr1-1 of *Arabidopsis thaliana* [36]. In our previous investigation, we have shown that exposure for 6 days to suboptimal temperature for tomato plant (M82) at a normal light intensity led to a reduction of oxygen evolution of detached leaves, although no significant changes in the ratio Fv/Fm were observed [27]. In the present study, the ratio Fv/Fm did not change significantly, but apparent ETR was influenced by low light intensity, better expressed at LT for Ailsa Craig. In tomato plants exposed to various combinations of low temperature and low light, Fang et al. [31] found that the ratio Fv/Fm could be equal to that of the control, but ETR for all treatments could not reach the control, suggesting that PSII reaction centers may be irreversibly damaged under these circumstances.

### 3.2. Photochemical Activity of PSI and PSII in Thylakod Membranes of Tomato Plants

The exposure to low temperature has been recognized as having one of the most significant environmental effects on plant physiological performance [37,38]. The interruption of photosynthetic electron transport, the carbon reduction cycle, and decreased stomatal conductance have all been implicated in the cold-stress-induced restriction of photosynthesis [1,39]. Low-light treatment for 5 days at NT resulted in a decrease of PSI- and PII-mediated electron transport rates in Ailsa Craig thylakoids and no restoration was observed during the recovery period. This inhibition reflected the effect of low light intensity to which the plants have to adapt. Our data showed that when LL was applied at LT, PSII was less inhibited in Ailsa Craig. The applied temperature (15/10 °C) was suboptimal but not extreme. No considerable effects could be expected and there was a balance between light absorption and metabolic reactions. It is interesting to note the observed differences in PSII inhibition between the wild type and the mutant: PSII in the mutant was similarly inhibited at both temperatures, whereas in the wild type, PSII inhibition was weaker at low temperatures compared to the optimal temperature. At present, we cannot propose an explanation but one could speculate that the possible configuration of β-carotene in reaction center proteins could influence in a different manner the stability of the complex.

### 3.3. Energy Distribution and Interaction between Both Photosystems, and PSII Antenna Size Altertaions

Higher plants have a special and extremely beneficial mechanism for regulating the effectiveness of photosynthetic reactions under various environmental factors (such as light, temperature, etc.), including adjustments of energy distribution between the two photosystems. At 77K, the chlorophyll fluorescence emission spectra contain two maxima at 685 nm and 735 nm and a shoulder (or peak depending on the plant species or treatment) at 695 nm. The bands at 685, 695 and 735 nm are attributed to the emission from the main pigment–protein complexes—F685 and F695 nm to PSII–LHCII complex, and F735 to PSI–LHCI complex (Figure S1). The detailed analysis of emission in the region 685–695 related more precisely the maxima to the emission from different complexes of PSII—emission at 685 nm to the PSII reaction center and CP43 and at 695 nm—to CP47. The intensities of the fluorescence emission peaks at 77K in the range of 685–695 nm and 735 nm were a reflection of the energy confined to PSI and PSII. It was evident from comparing Ailsa Craig to *tangerine* that the mutant exhibited a greater rise in F735/F685 after 5 days at LL-NT. This difference might have been caused by a reduction in PSII antenna or by an increase in the quantity of energy delivered to PSI as has been observed under the influence of stressors or during acclimation to changed environment in *Arabidopsis thaliana* [40,41] and tomato [42].

The F685/F695 ratio in Ailsa Craig revealed that, under LL-NT conditions, there was no change in the energy interaction between the PSII-CP43 and CP47 complexes, but the small increase in F685/F695 at LT most likely reflected some disruption of energy exchange between the two complexes. Emission of the PSII-CP43 complex in *tangerine* membranes was slightly lower after 5 days of LL-NT treatment compared to CP47, but the F685/F695 ratio was not affected by low-temperature treatment.

By examining the emission excitation spectra at 685 nm and 735 nm, it was possible to determine the participation of Chl a and Chl b molecules in the energy supply of the two photosystems and to asses any change in the antenna complexes [43]. Presented data showed that, at the control temperature, the participation of Chl b in the energy supply of PSII was increased relatively to Chl a. An increase of Chl b content as a response to low-light treatment has been observed in rice [6].

In order to follow possible changes in PSII antenna complexes we evaluated alterations in the abundance of CP43, CP47, Lhcb1 and Lhcb2 proteins during the treatment and recovery period. Under the LL treatment of Ailsa Craig plants, Lhcb1 and Lhcb2 decreased to the same extent at both temperatures and followed a similar trend. In the mutant, Lhcb2 was less affected than Lhcb1 and the decrease of Lhcb1 was more expressed at LT. Lhcb1 and Lhcb2 together with Lhcb3 are proteins that form the LHCII trimers. Lhcb1 and Lhcb2 are more abundant and although having nearly identical amino acid composition, the functional roles of Lhcb1 and Lhcb2 are different and complementary in respect of phosphorylation, state transitions and modulation of grana stacking [44]. The structure and organization of LHCII may be compromised at LL and LT treatment, and any difference in the pigment composition of Lhcb1 and Lhcb2 may account for their different sensitivity. Data available in the literature show very little differences in the pigment composition of the leaves of Ailsa Craig and *tangerine*^3138^, but there is no available data on the carotenoid composition in terms of *cis* and *trans* isomers [17,24]. Low-light treatment had a significant impact on the protein CP43 from the proximal antenna of PSII at both temperatures and in both plants; however, CP47 was less negatively impacted by low temperature in Ailsa Craig and in *tangerine*. During the plants’ recovery period, no restoration was observed in either Ailsa Craig nor in *tangerine*. It is possible that CP43’s dependence on D1 functioning and turnover accounts for its increased sensitivity.

### 3.4. Changes of Lipid Order of Thylakoid Membranes from Ailsa Craig and Tangerine

Our data showed relatively higher fluidity of thylakoid membranes from *tangerine* mutant (parameter P was 0.255 ± 0.013 for Ailsa Craig and 0.242 ± 0.012 for *tangerine*). It has been reported that the perturbations in carotenoid levels and composition can result in changes of lipids of plastadial membranes [45,46]. It has been suggested that α and β carotene directly affect membrane fluidity. The lipid component of thylakoid membranes is also influenced by environmental temperature; as the temperature decreases, plants tend to improve the membrane fluidity by raising the unsaturation levels of the lipid fatty acids [47]. The degree of unsaturation of fatty acyl chains of membrane lipids determines the chilling sensitivity of a plant and it has been shown that the unsaturation of membrane lipids of chloroplasts stabilizes the photosynthetic machinery against low-temperature photoinhibition in vivo in transgenic tobacco plants [48,49,50]. During treatment with LL-LT, a decrease in P (increase in fluidity) was detected for thylakoid membranes from Ailsa Craig and less expressed for *tangerine*. The fluidity of thylakoid membranes is of utmost importance for the effective interaction of electron transport carriers, the activity of many membrane-bound enzymes and its change is one of the mechanisms of adaptation to stressful conditions.

## 4. Conclusions

Up to now, the leaves of *tangerine* mutants did not attract attention, as in the presence of light, photoisomerization occurs and the role of CRTISO is not determinative. Despite the possibility of light-induced *cis-trans* isomerization of prolycopene to *all-trans*-lycopene, this photoisomerization was shown to occur in limited yield and to be initiated only in the presence of a triplet sensitizer [51]. Furthermore, *ccr2* mutants of *Arabidopsis thaliana,* grown in the light, show a reduction in lutein, suggesting that it is not excluded that β- and ε-cyclases differ in their requirements for CRTISO activity [52]. There may be other crucial roles for CRTISO in photosynthetic species given that cyanobacteria also appear to produce *trans* -lycopene via phytoene desaturase, _ζ -carotene desaturase and CRTISO while lacking both etioplasts and chromoplasts [53]. In the current study, we showed that the tomato *tangerine* mutant’s photosynthetic apparatus responds differently in comparison with Ailsa Craig to low-light and low-temperature treatment. While PSII activity in Aisla Craig was less reduced at LL-LT, it was inhibited to the same level in *tangerine* at both LL-NT and LL-LT. The fluidity of the *tangerine* thylakoid membrane was higher in comparison with Aisla Craig membranes. The abundance of the proximal antenna protein Lhcb2 was less affected by the LL-LT and LL-NT treatments of *tangerines*. The data presented indicated that the primary differences between the wild-type Aisla Craig and the mutant *tangerine,* with defective prolycopene isomerase, are mainly linked to PSII photochemical activity and its antenna complexes.

## 5. Materials and Methods

### 5.1. Plant Material

The *tangerine* mutant (LA3183) and its nearly isogenic WT “Ailsa Craig” (+/+) (LA2838A) were kindly provided by the Tomato Genetics Resource Center, Davis, CA, USA. Tomato plants were grown as described in Gerganova et al. [27]. Briefly, seeds were soaked on moist filter paper for 48 h at room temperature, transferred into perlite-containing soil and kept at 4 °C for 4 days. The plants were further grown under controlled conditions for 22 days: day/night cycle 16 h/8 h at temperature 24/22 °C and 250 μmol m^−2^ s^−1^ photon flux density (PFD). Fully developed tomato plants at the stage of 3rd leaf were treated for 5 days under different conditions—LL-NT (24/22 °C and 125 μmol m^−2^ s^−1^) and LL-LT (15/10 °C and 125 μmol m^−2^ s^−1^). After 5 days of treatment the plants were returned to normal conditions (NL-NT) and allowed to recover for 3 days, noted hereafter as 3 days R. Samples were taken at the beginning of each experiment (day 0), after 5 days of treatment and after 3 days of recovery. To ensure controlled conditions, all experiments were conducted under laboratory conditions using plant chambers.

### 5.2. Determination of Pigment Content

The pigment content of leaves Chl a, Chl b and Car was estimated in 80% acetone extract using the equations of Lichtenthaler [54], as described in Gerganova et al. [27].

### 5.3. PAM Chlorophyll a Fluorescence and Determination of Fv/Fm and ETR

In vivo chlorophyll a fluorescence was measured using a PAM (Pulse Amplitude Modulation) fluorimeter (H. Walz, Germany, model PAM 101–103) using detached dark-adapted (15 min) leaves. The initial fluorescence level (Fo) was registered at a 1.6 kHz frequency and a low-intensity measuring light of 0.120 μmol m^−2^ s^−1^ PFD. The maximal fluorescence level (Fm) in the dark-adapted state was measured after a white flash of 3000 μmol m^−2^ s^−1^ with a duration of 0.8 s. The maximal quantum yield of PSII was calculated as Fv/Fm = (Fm − Fo)/ Fm. The photosynthetic process was initiated by illumination for 5 min with actinic light of 250 μmol m^−2^ s^−1^ PFD corresponding to the plants’ light growth conditions and, every minute, a light saturation pulse was given for the determination of maximal fluorescence level in light adapted state (Fm’) and minimal fluorescence level in the light-adapted state (Fs) was measured after switching off the actinic light. The nomenclature of van Kooten and Snel [55] was used for the parameters of Chl fluorescence. The effective quantum yield of PSII was calculated as ΦPSII = (Fm’ − Fs)/Fm’. The relative electron transport rate (ETR) is the product of ΦPSII and photosynthetic photon flux density (PPFD) ETR = PAR × 0.84 × 0.5 × ΦPSII.

### 5.4. Isolation of Thylakoid Membranes

Thylakoid membranes from leaves of control, treated and recovered tomato plants were isolated, as described in Gerganova et al. [56]. The isolation was carried out at 4 °C under green light. The pigment content Chl (a+b) and Car in thylakoids was determined using the method and equations of Lichtenthaler [54], using extracts in 80% acetone.

### 5.5. Photochemical Activities of PSI and PSII in Isolated Thylakoid Membranes

For determination of the photochemical activities of both photosystems in thylakoid membranes exogenous electron donors and acceptors were applied [57,58]. PSII activity was evaluated by the oxygen evolution of thylakoids with a Clark-type electrode (DW1, Hansatech Instruments Ltd., Pentney, UK) in the presence of 0.4 mM 1,4-benzoquinone as an exogenous electron acceptor under saturating white light (1200 μmol m^−2^ s^−1^) provided by an LED source (halogen lamp JDR 230 V 75 W (Bingsheng Lighting Appliance Co. Ltd., Jiaxing, China)). The reaction medium for measurements of oxygen evolution contained 20 mM MES (pH 6.5), 0.33 M sucrose, 5 mM MgCl_2_ and 10 mM NaCl. The electron transport rate through PSI (from reduced DCPIP to methyl viologen (MV)) was measured by oxygen uptake in a reaction medium containing 20 mM Tricine (N- (Tri(hydroxymethyl) methyl)glycine) (pH 7.5), 0.33 M sucrose, 5 mM MgCl_2_, 10 mM NaCl, 0.4 mM DCMU (3-(3,4-dichlorophenyl)-1,1-dimethylurea), 0.5 mM NH_4_Cl, 5 mM NaN_3_ and reduced DCPIP (0.1 mM 2,6-dichlorophenol-indophenol and 4 mM Na ascorbate) as an artificial donor and 0.1 mM methyl viologen as an exogenous acceptor. Oxygen evolution and uptake were measured at 22 °C in thylakoid membranes corresponding to 25 µg Chl mL^−1^.

### 5.6. Low-Temperature (77 K) Fluorescence Measurements

Fluorescence emission and excitation spectra at 77 K (in liquid N, using a homemade Dewar flask) were recorded by a spectrofluorimeter (Jobin Yvon JY3, Division d’Instruments S.A., Longjumeau, France), as described by Faik et al. [58]. For the analysis of the emission and excitation spectra and for the calculation of the fluorescence ratios, Origin (ver. 6.0, Microcal Corp., Amherst, MA, USA, OriginLab Corporation, Northampton, MA, USA) was used. The emission spectra were recorded under excitation with 436 nm. The excitation spectra of fluorescence emitted from PSI and PSII were recorded at 735 and 685 nm in the red (610–700 nm) and Soret (410–500 nm) regions, respectively.

### 5.7. Steady-State Fluorescence Polarization Measurements

A fluorescent probe DPH (1,6-diphenyl-1,3,5-hexatriene) was used to determine the fluidity of the hydrophobic interior of biological membranes and especially of thylakoid membranes, as it tends to distribute evenly between all lipid domains and no energy transfer occurs between DPH and photosynthetic pigments. The fluidity of isolated membranes was estimated by measuring the degree of polarization of the steady-state fluorescence emitted from DPH at room temperature, as described previously [30]. DPH was added to a final concentration of 2.5 µM from a stock solution. Measurements were performed in the resuspending buffer (0.33 M sucrose, 5 mM MgCl_2_, 10 mM NaCl and 20 mM Tricine, pH 7.5) using a fluorimeter JASCO FP8300 (Jasco, Tokyo, Japan), equipped with polarization filters. Fluorescence was excited at 360 nm and registered at 450 nm at a chlorophyll concentration of the samples of 5 µg mL^−1^. The slit widths were 5 nm. The degree of polarization (P) was estimated using a formula described previously [28].

### 5.8. SDS–PAGE Electrophoresis and Western Blot

The alterations in photosystem II were evaluated using an analysis of CP43, CP47, Lhcb1 and Lhcb2 polypeptides. Proteins of thylakoid membranes from the controls and treated tomato plants grown under different combinations of temperature and light intensity were analyzed in a Laemmli SDS–PAGE system. The polyacrylamide concentrations of the stacking and resolving gels were 4 and 12%, respectively, with 4 M urea added to the resolving gel. The samples were incubated with a sample buffer (3:1) in the dark for 1 h at room temperature. Equal volumes of thylakoid membranes, corresponding to 3 µg Chl were loaded in every line. The proteins were transferred from gel to nitrocellulose membranes, and proteins were probed with antibodies for CP43 (AS11 1787 at a dilution of 1:3000, Agrisera, Vännäs, Sweden), CP47 (AS04 038 at a dilution 1: 2000, Agrisera, Vännäs, Sweden), Lhcb1 (As01 004 at a dilution of 1:2000, Agrisera, Vännäs, Sweden) and Lhcb2 (AS01 003 at a dilution 1:5000, Agrisera, Vännäs, Sweden). The Alkaline Phosphatase Conjugate Substrate Kit (Bio-Rad, Hercules, CA, USA) with goat anti-rabbit (GAR) secondary antibodies was used for the development of the blocked membranes, which were quantified with ImageJ software. Immunoblotting was repeated 3 times with thylakoid membranes from two different experiments.

### 5.9. Statistics

In all graphics, data were presented as mean values (±SE) from two independent experiments with 4 parallel samples at every time point. Changes in investigated parameters of Ailsa Craig and *tangerine* mutant as affected by treatment with LL in combination with NT or LT were statistically evaluated using Fisher’s least-significant difference test at *p* ≤ 0.05 following multifactor ANOVA analysis. A statistical software package (Statgraphics Plus, version 5.1 for 735 Windows, The Plains, VA, USA) was used.

## Figures and Tables

**Table 1:** Photosynthetic pigment content (Chl (a+b) and Car) in Ailsa Craig and carotenoid mutant *tangerine* after treatment of plants for 5 days with low light (LL) in combination with optimal (NT) or low (LT) temperature and after recovery period of 3 days. Data were presented as (mg pigment g-1 FW). Mean values ± SE were calculated from two independent experiments with 4 parallel samples at each time point (*n* = 8). Significant differences between values at *p* < 0.05, as estimated by Fisher’s LSD test of multifactor ANOVA analysis, were indicated with different letters.

	Chl (a+b)	Chl a/b	Car
Ailsa Craig			
0 days	2.24 ± 0.03 a	3.28 ± 0.10 a	0.53 ± 0.01 a
5 d NT-LL	2.06 ± 0.17 a	3.21 ± 0.11 a	0.43 ± 0.02 c
5 d LT LL	1.72 ± 0.09 b	3.21 ± 0.15 a	0.41 ± 0.01 c
3 d R NT-LL	2.12 ± 0.16 a	3.31 ± 0.13 a	0.47 ± 0.02 b
3 d R LT-LL	1.77 ± 0.07 b	3.29 ± 0.18 a	0.41 ± 0.01 c
*Tangerine*			
0 days	2.11 ± 0.09 a	3.59 ± 0.03 ac	0.50 ± 0.02 a
5 d NT-LL	1.83 ± 0.05 bc	3.46 ± 0.04 bc	0.46 ± 0.01 bc
5 d LT LL	1.69 ± 0.08 c	3.21 ± 0.08 c	0.40 ± 0.01 c
3 d R NT-LL	1.88 ± 0.12 b	3.52 ± 0.15 a	0.53 ± 0.03 a
3 d R LT-LL	1.79 ± 0.06 bc	3.29 ± 0.14 bc	0.41 ± 0.01 bc

## Data Availability

Data are contained within the article and supplementary materials.

## Supplementary Materials

The following supporting information can be downloaded at https://www.mdpi.com/article/10.3390/plants12163000/s1. Figure S1: Typical 77K emission and excitation spectra of thylakoid membranes, isolated from control and treated for 5 days at LT-LL plants Aisla Craig and tangerine; Figure S2: Representative Western blots of PSII-LHCII-related proteins CP47, CP43, Lhcb 1 and Lhcb2 in thylakoid membranes isolated from control, treated and recovered plants—Aisla Craig and tangerine.
