# Supplementary material for: Effect of Low Light on Photosynthetic Performance of Tomato Plants—Ailsa Craig and Carotenoid Mutant Tangerine
Source: Plants (Basel). 2023 Aug 20;12(16):3000. doi: 10.3390/plants12163000 (PMC10459318; doi:10.3390/plants12163000)
Supplement: Supplementary file 1 [file plants-12-03000-s001.zip › Suppl mat Plants 2023.pdf]

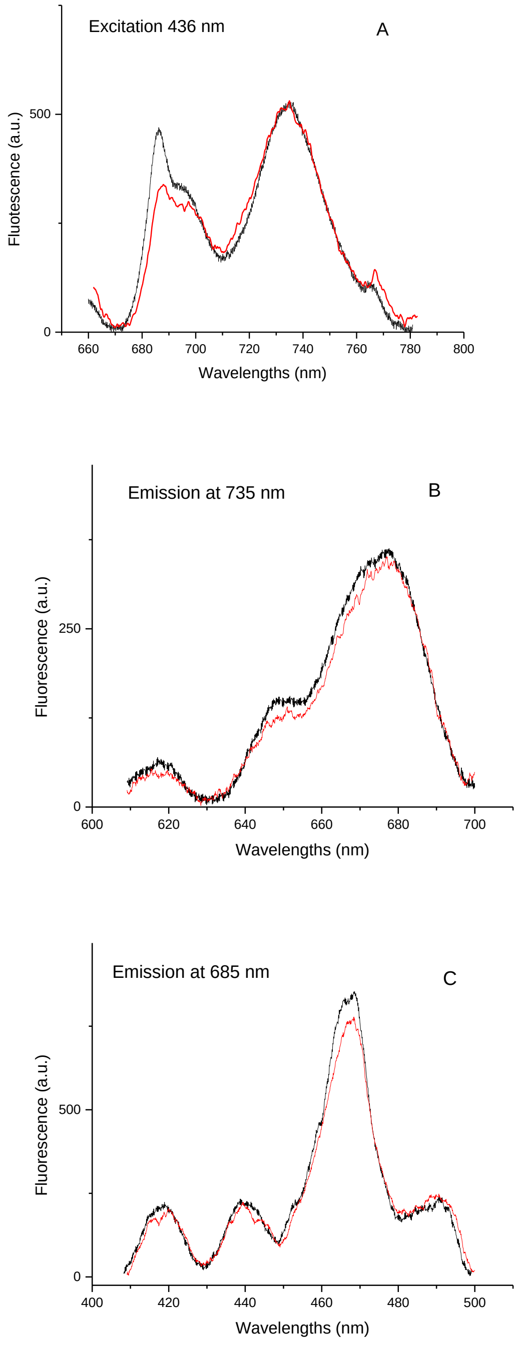

Figure S1. Typical 77K emission (A) and excitation (B, C) spectra of thylakoid membranes, isolated from control (black lines) and treated or 5 days at LT-LL (red lines) plants Aisla Craig (A, B) and *tangerine* (C). A - Excitation wavelength - 436 nm; B - excitation spectra of fluorescence at 735 nm (emitted from PSI-LHCI complexes); C - excitation spectra of fluorescence at 685 nm (emitted from PSII–LHCII complexes).

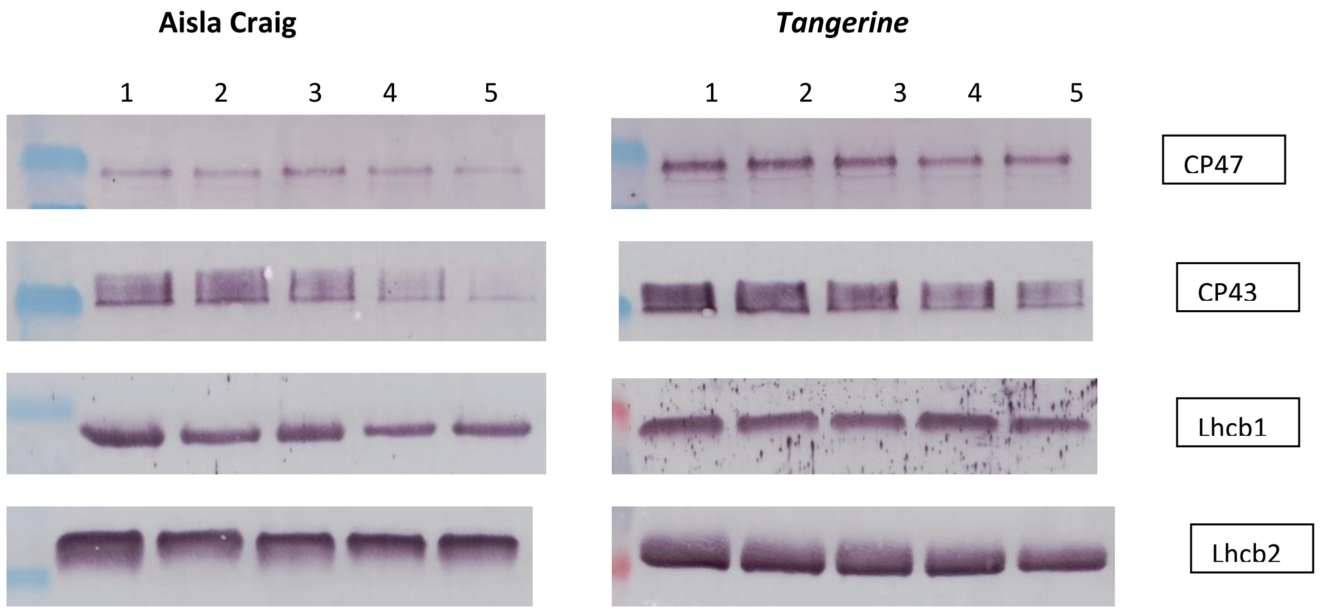

Figure S2. Representative Western blots of PSII-LHCII - related proteins - CP47, CP43, Lhcb1 and Lhcb2 in thylakoid membranes isolated from control, treated and recovered plants – Aisla Craig and *tangerine*. Path numbers: 1 – 0 days; 2 – 5 days NT-LL; 3 – 5 days LT-LL; 4 – 3 days R NT-LL; 5 – 3 days R LT-LL.
